# Spectrum of clinical features and neuroimaging findings in acute cerebral infarction patients with unusual ipsilateral motor impairment– a series of 22 cases

**DOI:** 10.1186/s12883-019-1516-y

**Published:** 2019-11-12

**Authors:** Zhe-Ren Tan, Chen Zhang, Fa-Fa Tian

**Affiliations:** Department of Neurology, Xiangya Hospital, Central South University, 87 Xiangya Road, Changsha, 410008 China

**Keywords:** Ipsilateral stroke, Cerebral infarction, Cerebrovascular disease, Neuroimaging, Hemiparesis

## Abstract

**Background:**

Cerebral infarction occurs when the arteries to brain are obstructed, and motor impairment contralateral to responsible lesion is commonly recognized. Few studies have profiled the characteristics of cases with ipsilateral motor impairment. We sought to characterize clinical features of patients with motor dysfunction caused by ipsilateral ischemic stroke.

**Methods:**

We retrieved and analyzed the medical data for patients with ipsilateral cerebral infarction. Patients were regarded as having ipsilateral cerebral infarction if motor impairment is ipsilateral to recent stroke lesions.

**Results:**

Only 22 patients with unusual ipsilateral cerebral infarction were included in this study. Ipsilateral limb paralysis was observed in all cases, and one case showed central facioplegia. Majority of patients with limb paralysis (90.9%, 20/22) presented with mild muscle strength deficits (MRC grading of 4 or more). Most of the patients (72.7%, 16/22) had a past history of stroke, and previous strokes were contralateral to the side of the recent stroke in 14 out of 16 patients (87.5%). No history of stroke or cerebral injury was identified in seven patients. With aspect of MRI findings, recent infarct lesions of all cases were located along the corticospinal tract.

**Conclusions:**

History of stroke plays an important role in the pathogenesis of ipsilateral motor impairment, and cortical reorganization in the unaffected hemisphere may contribute to the compensation of motor function after stroke. Besides that, some cases with first stroke may be due to impairment of ipsilateral uncrossed corticospinal fibers.

## Background

Previous neuroanatomic studies have firmly demonstrated that the primary motor cortex predominantly innervates the contralateral half of the body. Almost all patients with supratentorial stroke involving the pyramidal tract exhibit contralateral motor dysfunction. Conversely, more and more cases of ipsilateral stoke or deterioration of hemiparesis ipsilateral to the cerebral injury are reported [1–4], One previous study focused on ipsilateral hemiparesis in ischemic stroke patients found a high proportion of history of stroke contralateral to the recent stroke in cases of ipsilateral hemiparesis [5], which indicating the need to investigate unusual ipsilateral stroke for exploring the mechanism of post-stroke compensatory motor function, such as the enhanced pyramidal tract [1, 6]. increased cerebral blood flow [7, 8] and increased activation of motor regions [9, 10] in unaffected hemisphere after stroke. However, severe case reports of stroke leading to ipsilateral motor weakness are published but there is no study that summarizes the clinical characteristics and neuroimaging findings of cerebral infarction patients with unusual ipsilateral motor impairment of limb and cranial nerves.

In present study, we sought to investigate and characterize the clinical features and magnetic resonance imaging (MRI) findings in cerebral infarction patients with ipsilateral motor impairment.

## Methods

We retrieved the medical data for ipsilateral cerebral infarction patients treated between Jan 2011 and June 2018 in the Department of Neurology, Xiangya Hospital, Central South University, Changsha, China.

Patients were diagnosed as ipsilateral cerebral infarct and included in present study if they satisfied the following criteria: (1) For each case, the diagnosis of recent cerebral infarct was confirmed by complete clinical assessment and MRI. (2) Patients who experienced confirmed motor impairment due to the recent cerebral infarct. (3) The responsible lesions of patients with limb paralysis were located above the decussation of pyramid and ipsilateral to their motor impairment. (4) The responsible lesions of patients with central cranial nerve paralysis were located above the brainstem and ipsilateral to their motor impairment. (5) Patients who underwent MRI and MRI radiographs of all patients show recent hyperintense lesions on T2-weighted images and diffusion-weighted imaging (DWI) which is ipsilateral to recent motor dysfunction. (6) Medical data are complete and available. (7) Informed consent was obtained from patients or their guardians.

The following were the exclusion criterias (1) Patients with difficulty in explaining their past and recent motor deficits.

Demographic data, clinical characteristics, and neuroimaging findings were recorded.. The neuroimaging scan was performed on a 1.5 T MRI scanner (GE Healthcare, Milwaukee, Wisconsin).

## Results

Twenty-two acute cerebral infarction patients (16 men and 6 women) with unusual ipsilateral motor impairment and brain MRI records available for review were found and included in this study. Average age of onset was 63.4 (range 48–83) years.

### Clinical presentation

Ten patients had right side ipsilateral paralysis, 11 patients had left side ipsilateral paralysis and one patient had bilateral paralysis. The most frequent symptom was ipsilateral limb paralysis, which was observed in all cases. The other unusual symptom was central facioplegia (1 case).

Majority of patients with limb paralysis (90.9%, 20/22) presented with mild muscle strength deficits (MRC grading of 4 or more), two patients (9.1%, 2/22) presented with moderate muscle strength deficits (MRC grading of 3) and no patient presented with severe muscle strength deficits (MRC grading of 2 or less).

Three patients presented with hyperreflexia in paralyzed limbs and four patients presented with hyporeflexia in paralyzed limbs. Five patients presented with pathological reflexes in paralyzed limbs.

The most of the patients (72.7%, 16/22) had a history of stroke (cerebral infarction in 14 and cerebral hemorrhage in two), and previous strokes were contralateral to the side of the recent stroke in 14 out of 16 patients (87.5%). No history of stroke or old lesions were identified in seven patients. Demographics and clinical characteristics of all cases are listed in Table 1.

**Table 1:** Clinical profile in cerebral infarction patients with unusual ipsilateral motor impairment

No.	Past stroke		Recent CI						
	Type and Main symptoms	Interval between strokes	Side and type of paralysis	MRC grading (arm/leg)		DTR (arm/leg)		Pathological reflex (arm/leg)	
				Ipsi	Contr	Ipsi	Contr	Ipsi	Contr
1	CI, LP(R)	3 months	R/LP	4/4	5/5	++/++	++/++	−/−	−/−
2	–	–	L/LP	4/4	5/5	++/++	++/++	−/−	−/−
3	CI, LP(R)	3 years	L + R/LP	4/4	4/4	++/++	++/++	−/−	−/−
4	CH, LP(L)	3 months	L/LP	4/4	5/5	++/++	++/++	−/−	−/−
5	CI, LP(L)	2 years	L/LP	4/4	5/5	++/++	++/++	−/−	−/−
6	CI, LP(R)	1 years	L/LP	4/4	5/5	++/++	++/++	−/−	−/−
7	CI, LP(R)	2 years	R/LP	4/4	5/5	++/+++	++/++	−/−	−/−
8	–	–	R/LP	4/4	5/5	+/+	++/++	−/−	−/−
9	CI, LP(R)	1 years	R/LP	4/4	5/5	++/++	++/++	−/+	−/−
10	CI, LP(R)	1 years	L/LP	4/4	4/4	++/++	++/++	−/−	−/−
11	–	–	L/LP	4/4	5/5	+/+	++/++	−/+	−/−
12	CI, LP(R)	5 years	R/LP	4/4	5/5	+/+	++/++	−/−	−/−
13	CI, LP(R)	2 years	R/LP	3/4	5/5	++/++	++/++	−/+	−/−
14	–	–	L/LP	4/4	5/5	++/++	++/++	−/−	−/−
15	CH,LP(L)	3 years	L/LP	4/4	5/5	++/++	++/++	−/−	−/−
16	CI, HH(R)	5 years	R/LP	4/4	5/5	++/++	++/++	−/+	−/−
17	CI, LP(L)	8 years	L/LP	4/4	5/5	++/++	+/++	−/−	−/−
18	–	–	L/LP + CF	4/4	5/5	++/++	++/++	−/−	−/−
19	–	–	R/LP	4/4	5/5	++/++	++/++	−/−	−/−
20	CI, LP(R)	4 years	R/LP	4/4	5/5	+++/++	++/++	−/−	−/−
21	CI, LP(R)	1 year	R/LP	3/3	5/5	+++/+++	++/++	−/+	−/−
22	CI, LP(L)	3 years	L/LP	4/4	5/5	+/+	+/+	−/−	−/−

*CI* cerebral infarction, *CH* cerebral hemorrhage, *M* male, *F* female, *R* right, *L* left, *LP* limb paralysis, CF central facioplegia, *HH* Homonymous Hemianopia, *MRC* manual motor power test using Medical Research Council grading (arm/leg), *DTR* deep tendon reflex, *Ipsi* ipsilateral side to recent lesion, *Contr* contralateral side to recent lesion

### MRI findings

**Fig. 1:**
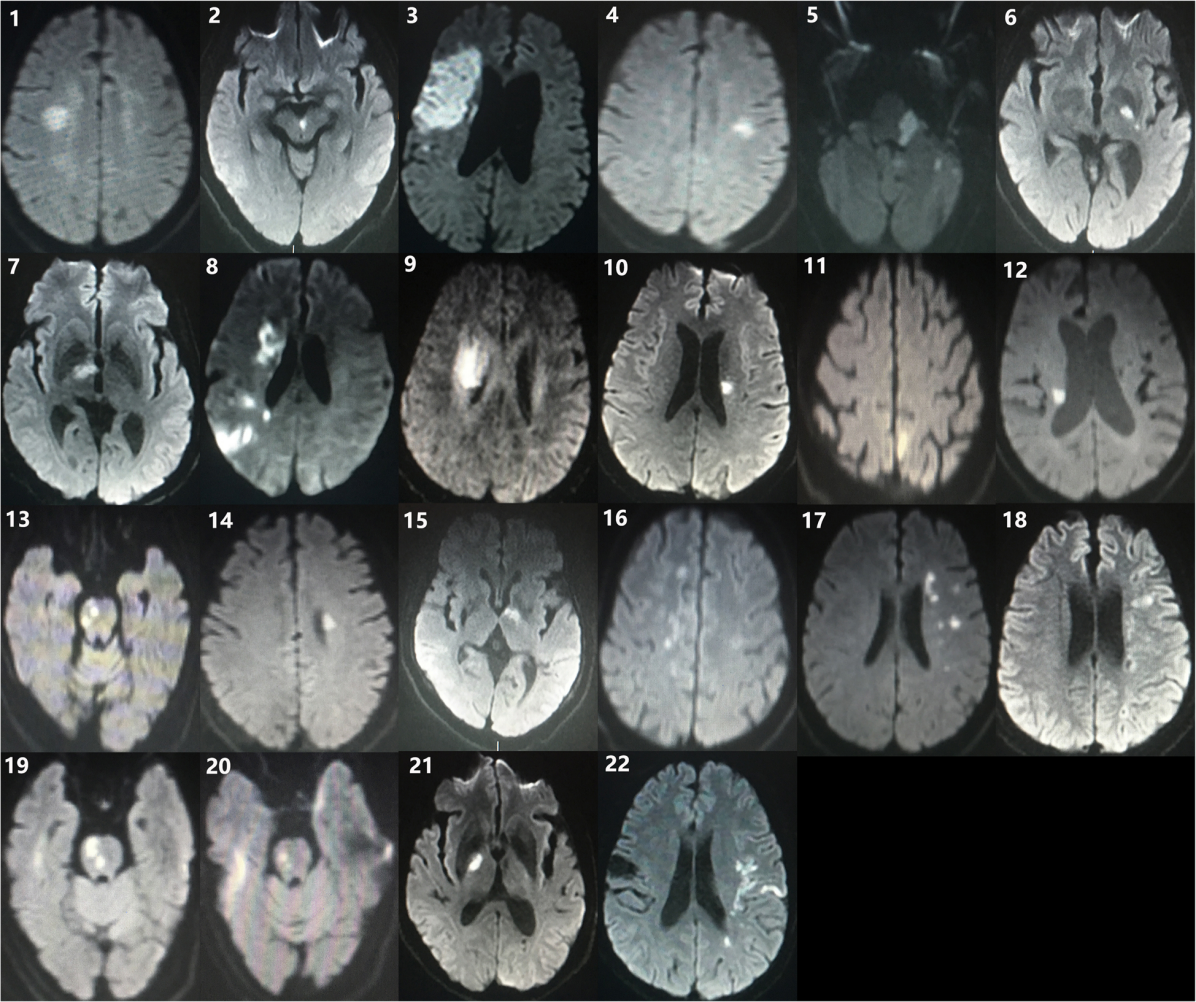
MRI radiographs of patients show responsible lesions of recent stroke on DWI

**Fig. 2:**
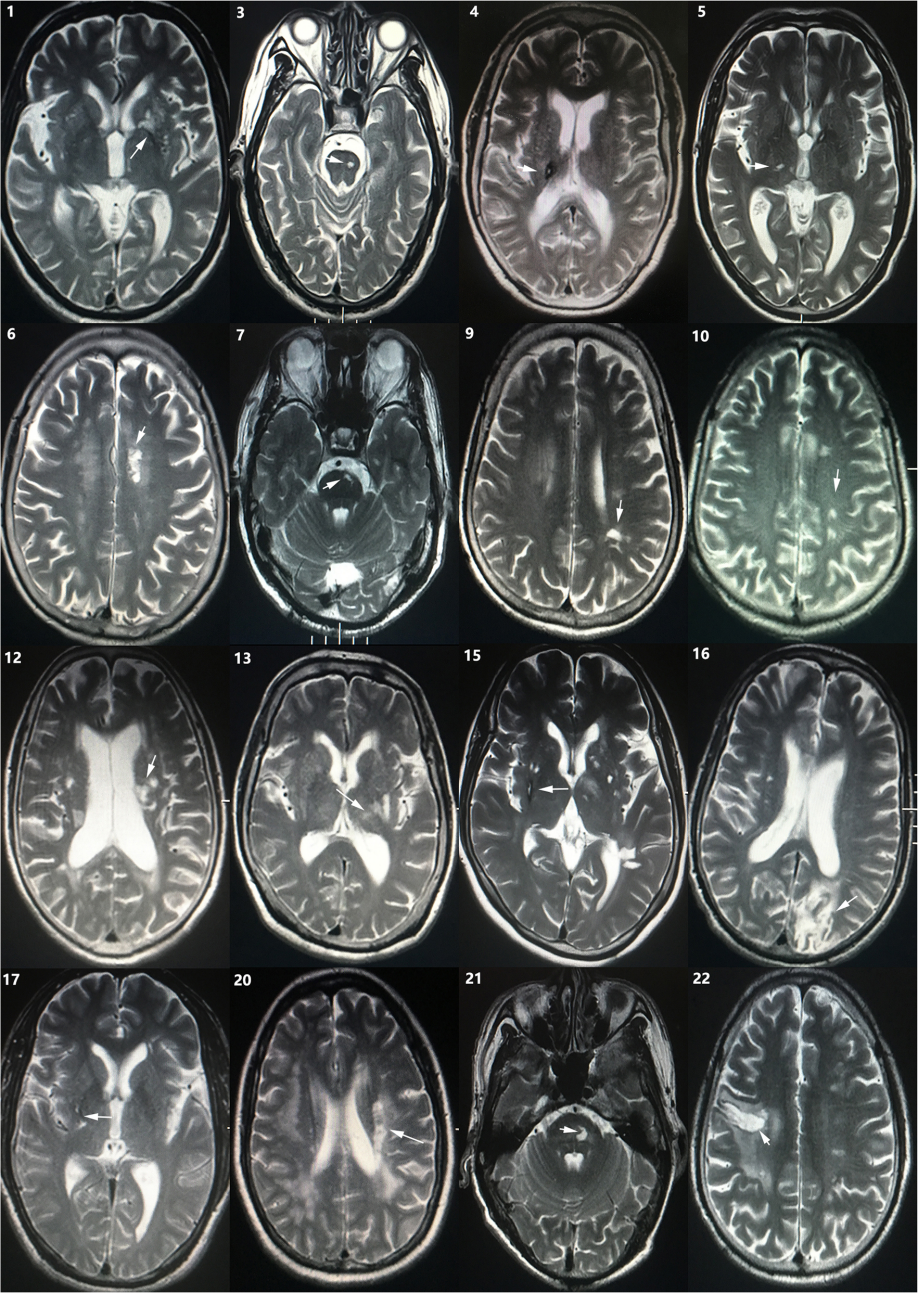
MRI radiographs of patients show responsible lesions of past stroke on T2-weighted images

Brain MRI was performed in all patients. The interval from onset to performing MRI was less than 15 days in most cases (19/22) in our study. The majority of responsible lesions for motor deficits were located in corona radiate(*n* = 10, 45.5%), internal capsula (*n* = 5, 22.7%), pons(*n* = 4, 18.2%), pedunculus cerebri(*n* = 1, 4.5%), middle frontal gyrus(*n* = 1, 4.5%), superior parietal lobule(*n* = 1, 4.5%). infract lesions of all cases are likely to involve pyramidal tract. All patient with the history of stroke had obvious old infarct lesions contralateral to neurological deficit in past stroke. No patient was reported to have cerebral malformations, such as arteriovenous malformation, Dandy-Walker malformation orogenesis of the corpus callosum, etc. Neuroimaging findings of all cases are summarized in Table 2 and Figs. 1 and 2.

**Table 2:** Neuroimaging finding in cerebral infarction patients with unusual motor impairment

No.	Past stroke	Recent CI		
	Location of lesion	Location of lesion	Malformation of brain	Dominant hemisphere
1	L BG	R CR	No	R
2	–	L PC	No	R
3	L Po	R FL,TL,PL	No	R
4	R T	L CR	No	R
5	R T	L Po, Ce	No	R
6	L CS	L BG	No	R
7	L Po	R BG	No	R
8	–	R FL, TL, PL,CR	No	R
9	L CR	R CR	No	R
10	L CS,PL	L BG	No	R
11	–	L GC, OL, PL,BP	No	R
12	L CR	R CR	No	R
13	L BG,FL	R Po	No	R
14	–	L CR	No	R
15	R BG	L BG	No	R
16	L OL	R GC, CR	No	R
17	R BG	L CR	No	R
18	–	L CR	No	R
19	–	R Po	No	R
20	L CR	R Po	No	R
21	L Po	R BG	No	R
22	R FL,PL	L CR FL,PL	No	R

*L* left, *R* right, *PO* pons, *Ce* cerebellum, *CR* corona radiate, *BG* basal ganglia, *BP* brachium pontis, *GC* gyrus cinguli, *CS* centrum semiovale, *PC* pedunculus cerebri, *FL* frontal lobe, *TL* temporal lobe, *PL* parietal lobe, *OL* occipital lobe, *T* thalamus

## Discussion

Unusual ipsilateral motor impairment after a cerebral stroke has rarely been reported. In the present study, we summarized the clinical features of ipsilateral cerebral infarct as small lesions in the MRI findings, a high proportion of hemiparesis, and the history of strokes contralateral to the recent strokes.

In our study, most cases demonstrated small patchy lesions (Fig. 1), and the recent responsible lesions located along pyramidal tract, which is consistent with findings of previous studies [5]. However, only patient 3 shows bilateral hemiparesis due to unilateral stroke, no obvious motor deficits contralateral to infarct were observed in the other 21 cases. A possible explanation for this phenomenon is that uncrossed and crossed corticospinal tracts (CST) might be located closely but clearly separated, and a small infarct is often asymptomatic, the lesion is so small that the direct damage to CST is limited, only uncrossed corticospinal tract is involved in those cases. If the responsible lesions are more extensive, it may cause bilateral paralysis like patient 3. Besides that, the physicians may be more concerned about symptoms contralateral to responsible lesions based on common sense and clinical experience thus unusual ipsilateral motor impairment may be neglected. Significant high proportion of mild muscle strength deficits (90.9%, 20/22) were also observed in the present study. Small lesion and prevailing influence of the lateral CST projection contralateral to recent infarct may limit the deficit of motor function.

Consistent with previous studies [5, 11], most patients in our study (72.7%, *N* = 16) showed history of stroke and it caused limb paralysis contralateral to responsible lesions. Besides that, previous strokes were contralateral to the side of the recent stroke in 14 out of 16 patients (87.5%), suggesting that recurrent deficit occurring in the same limb from previous stroke in those cases, and past strokes or brain injury contralateral to recent strokes might be associated with the pathogenesis of motor deficits ipsilateral to recent lesions. In previous study conducted by Weiller, et al. [7], regional cerebral blood flow was significantly increased in specific cortical areas of unaffected hemisphere following recovery from motor strokes; In a case reported by Ago, et al., only left motor cortex was activated by the paretic left hand movement after the first stroke, which suggested that cortical reorganization and intensification of the uncrossed CST in unaffected hemisphere occurred in the recovery process [1]. In fact, previous studies have identified cortical reorganization within the motor areas of the unaffected hemisphere related to post-stroke motor recovery [8–10], it might be one of the main mechanisms for unusual ipsilateral stroke.

Seven patients without history of stroke or cerebral injury and two patients whose recent lesions were on the same side as the previous stroke lesions were observed in our study, so that it cannot be explained by cortical reorganization. In the study conducted by Lacroix, et al., the terminals of marked CST were measured in gray matter of spinal cord, about 10% of CST axons terminating ipsilateral to the cortical origin were observed. Besides that, these axons were found to terminate mainly in close proximity to ipsilateral motor neurons, which demonstrated the substrate for cortical activation of ipsilateral muscle groups [12]. So that impairment of uncrossed CST originating from motor cortex could cause ipsilateral motor dysfunction.

In fact, several previous studies had reported patients without congenital decussation of corticospinal tract, but the lack of crossing fibers of those patients often was associated with congenital malformations, such as horizontal gaze palsy with progressive scoliosis (HGPSS), lissencephaly or Dandy Walker malformations [11, 13–16]. In a literature review of ipsilateral hemiparesis in stroke [5], three patients (18%/*n* = 17) showed structural defect, such as HGPSS and corpus callosum agenesis. In contrast, a remarkable finding in our cohort of 22 patients was the absence of congenital malformation. It seems that the proportion of patients with congenital malformations in the cases of ipsilateral stroke may be overestimated.

The corticonuclear tract is one part of the pyramidal tracts, it innervates cranial motor nuclei bilaterally with the exception of the lower part of facial nuclei and cranial nerve XII which are innervated only unilaterally. In present study, we found a case of supratentorial stroke that show central facioplegia ipsilateral to fresh high T2 and DWI signals in corona radiate and no history of brain injury and congenital malformations were recorded, similar cases have never been reported before. A possible explanation for occurrence of ipsilateral central facioplegia in this case is the presence of uncrossed corticonuclear tract. Alurkar et, al reported an ipsilateral stroke patient without any brain malformation and confirmed that motor cortex on the right controlled ipsilateral side was due to uncrossed pyramidal tracts using Diffusion Tensor Imaging (DTI) tractography [17].

The limitation of our study should be noted. We cannot confirm the type of pyramidal tract accurately without using DTI, which is meaningful for evaluating the path of pyramidal tract. Further studies are required to verify our speculation about the features reported in our cases and to determine the mechanism of cortical reorganization in the occurrence of ipsilateral stroke.

## Conclusion

In the present study, A high proportion of stroke history was observed, which means that history of stroke or cerebral injury plays an important role in the pathogenesis of ipsilateral motor impairment, and cortical reorganization in the unaffected hemisphere may contribute to the compensation of motor function after stroke. Besides that, some cases with first stroke may be due to impairment of ipsilateral uncrossed corticospinal tract.

## Data Availability

The data that support the findings of this study are available from the corresponding author via E-mail upon reasonable request.

## Abbreviations

CST: Corticospinal tracts
DTI: Diffusion Tensor Imaging
DWI: Diffusion-weighted imaging
HGPSS: Horizontal gaze palsy with progressive scoliosis
MRC: Manual motor power test using Medical Research Council grading
MRI: Magnetic resonance imaging
